# Injurious Fall in an Elderly Chronic Heroin Addict During a Syncopal Episode

**DOI:** 10.7759/cureus.37736

**Published:** 2023-04-17

**Authors:** Essence Maita, Girisha Bodavula, Aminat Bello, Rody Chehade, Sandesh Kuchipudi

**Affiliations:** 1 Internal Medicine, Saint James School of Medicine, Saint Vincent, VCT; 2 Internal Medicine, St. George's University School of Medicine, University Centre Grenada, GRD; 3 Internal Medicine, Loretto Hospital, Chicago, USA

**Keywords:** emergency medicine and trauma, drug addiction, fall assessment, geriatric injuries, opioid, syncopal episode, unexplained syncope, heroin, elderly falls, fall prevention

## Abstract

The risk factors and related negative outcomes associated with falls in the elderly population have been widely researched. Falls in the elderly population can lead to decreased independence and an increased risk of morbidity and mortality. Concomitant factors that can increase the risks of falls in the elderly include polypharmacy, vision impairment, syncope, hyporeflexia, and drug use. Presented is the case of a 79-year-old African American female who arrived at the emergency department after experiencing a syncopal episode at her home. The episode resulted in a non-fatal injurious fall. This case report examines the relationship between chronic drug use in an elderly patient and its predilection for syncopal episodes, which led to a non-fatal injurious fall.

## Introduction

According to the CDC, each year millions of elderly people, ages 65 and older, fall [1]. Falls in the elderly population can be attributed to numerous multifactorial causes but ultimately have similar detrimental outcomes for this group. In addition to the possible fatal outcomes of falls, many falls can be linked to non-life-threatening injuries that can eventually lead to increases in morbidity and mortality. Falls can also have a psychological effect on elderly individuals who become fearful of falling which negatively affects their mobility. This decrease in independence may result in prolonged disability and the development of declining mental and physical health [2].

Two risk factors pertinent to this case of an elderly fall include drug use and syncope. Opioid abuse, specifically heroin, has been endemic to many communities in the United States beginning in the early 1970s. Since then, there have been several waves of increased use. As these previous young adult populations age, the growing number of elderly addicted individuals has increased [3].

Heroin is an opioid drug made from morphine. There are various ways people can use heroin. Heroin can be injected, snorted, sniffed, or smoked. When heroin is injected intravenously it enters the brain at a rapid pace and acts on mu-opioid receptors which control pain, pleasure, heart rate, and breathing among other things [4]. Heroin overdose can lead to a slow respiratory rate. Lack of oxygen or blood flow to the brain can lead to syncope. Heroin addicts are known to experience syncopal episodes while using illicit drugs [5]. Syncope is defined as a sudden loss of consciousness associated with an inability to maintain postural tone, with immediate or spontaneous recovery. There is an increase in the incidence of syncope after age 70 [6]. Syncope in the elderly population is increasingly becoming a differential diagnosis for unexplained falls [7].

## Case presentation

A 79-year-old African American female presented to the emergency department with complaints of a head injury after a fall. The patient reported using heroin prior to the fall. The patient reported that she has used heroin off and on for the past 50 years and consistently for the last 10 years. She denied any other illicit drug use. She reported that she passed out and does not know exactly how the fall occurred. She also stated that as she spontaneously regained consciousness, she noticed a laceration to her left ear and presented to the emergency room for assistance. She stated that she does not have a power of attorney or guardian. The patient reported that she lives alone in a senior building. The also stated that she was at home alone at the time of her fall. She stated that her granddaughter acts as her caregiver. The patient reported that her granddaughter assists her with her activities of daily living approximately five days per week. She also stated that she previously used a walker for assistance with ambulation. While in the emergency department, she reported that she rates the pain in her head and neck area as a 5/10 on the pain scale. She denied any other neurological symptoms related to head injury including; weakness, numbness, incoordination, change in speech, confusion, or seizures.

The patient was admitted to the internal medicine unit for continued observation and monitoring. A physical exam was conducted. Her general appearance was cooperative and she showed no signs of acute distress. Her lungs were clear to auscultation, with normal air movement. Cardiovascular findings were regular rate, normal S1, normal S2, and no murmurs, gallops, or rubs. Her abdomen was soft, with no tenderness, no hepatosplenomegaly, and no masses. There was no clubbing, cyanosis, no edema seen in her extremities. The patient's past medical history included hypertension, arthritis, and gastroesophageal reflux disease. The patient denied any past surgical history. The patient reported that she was allergic to penicillin. CT scan of the head/brain without contrast was indicated due to a fall/loss of consciousness. Results of the CT scan showed there was no acute intracranial hemorrhage, mass, or acute ischemia. MRI of the brain without contrast was also indicated due to a syncopal episode. MRI findings showed that there was no acute intracranial hemorrhage, no midline shift, herniation, or hydrocephalus. The laceration to the patient's left ear was noted to be a superficial laceration to the posterior ear fold. Pressure and dressing were applied to the affected area of the patient's left ear. The laceration was further irrigated with copious amounts of saline and repaired with Dermabond. The patient's condition improved steadily throughout her seven-day inpatient course. 

**Table 1:** Patient vitals on admission to the hospital.

Patient Vitals				
Temperature (Reference range: 36.1°C-37.2°C)	Pulse (Reference range: 60-100 beats/minute)	Respiratory Rate (Reference range: 12-16 breaths/minute)	Blood Pressure (Reference range: Systolic: <120 mm Hg Diastolic: <80 mm Hg)	Oxygen Saturation (Reference range: 95-100%)
36.6°C	64 bpm	17 BPM	115/66 mm Hg	97%

**Figure 1:**
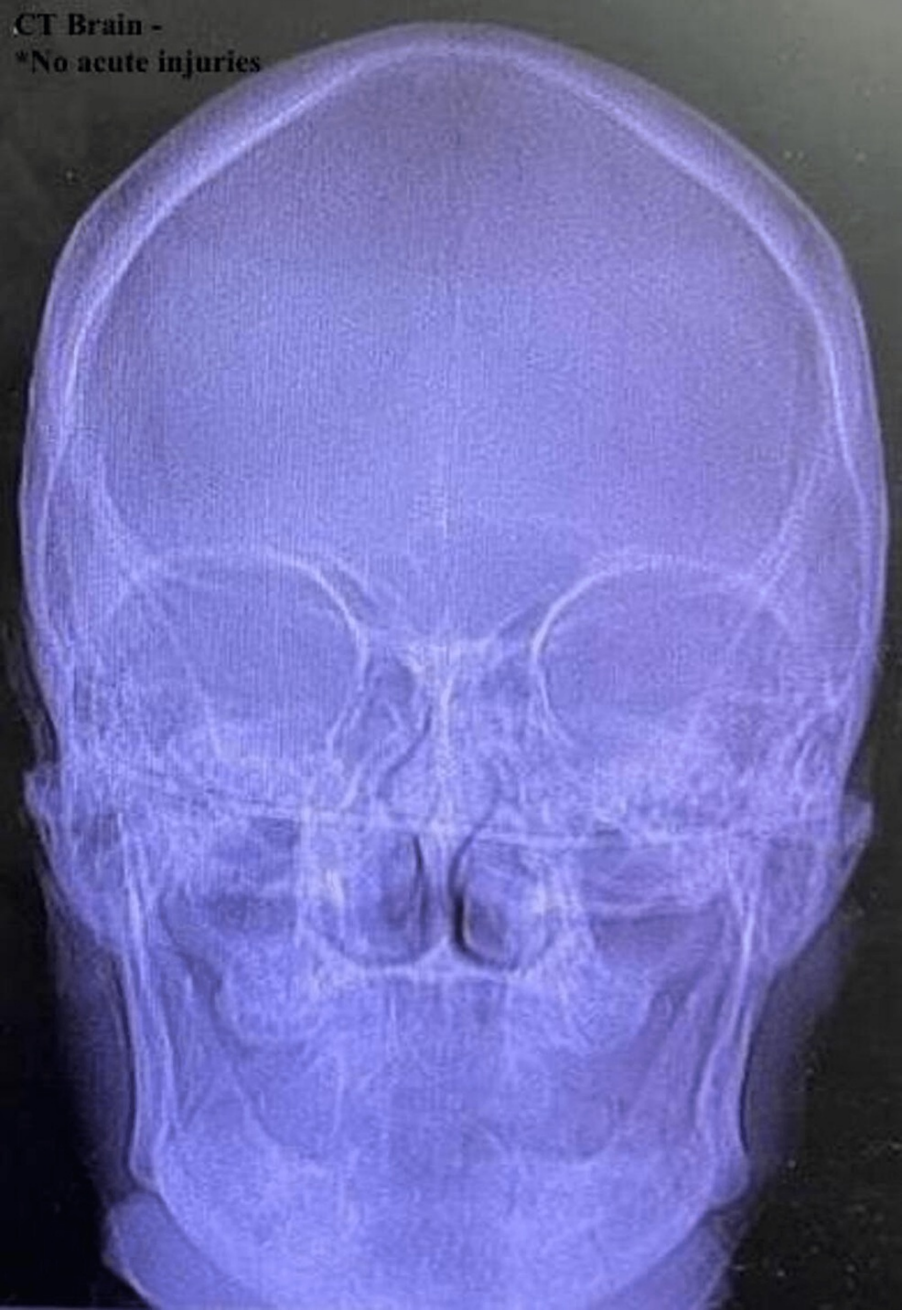
CT Brain Indication for the procedure: loss of consciousness, fall. Technique: CT of the brain without contrast. Exam date: 01/10/2023. CT findings: There was no intra or extra-axial hemorrhage, acute large vascular distribution infarct, or intracranial mass lesion. No hydrocephalus. The sella and suprasellar region were unremarkable. No cerebellar tonsillar herniation. Orbits were without acute abnormality. Mastoid air cells were clear. The scalp and calvarium were intact.

**Figure 2:**
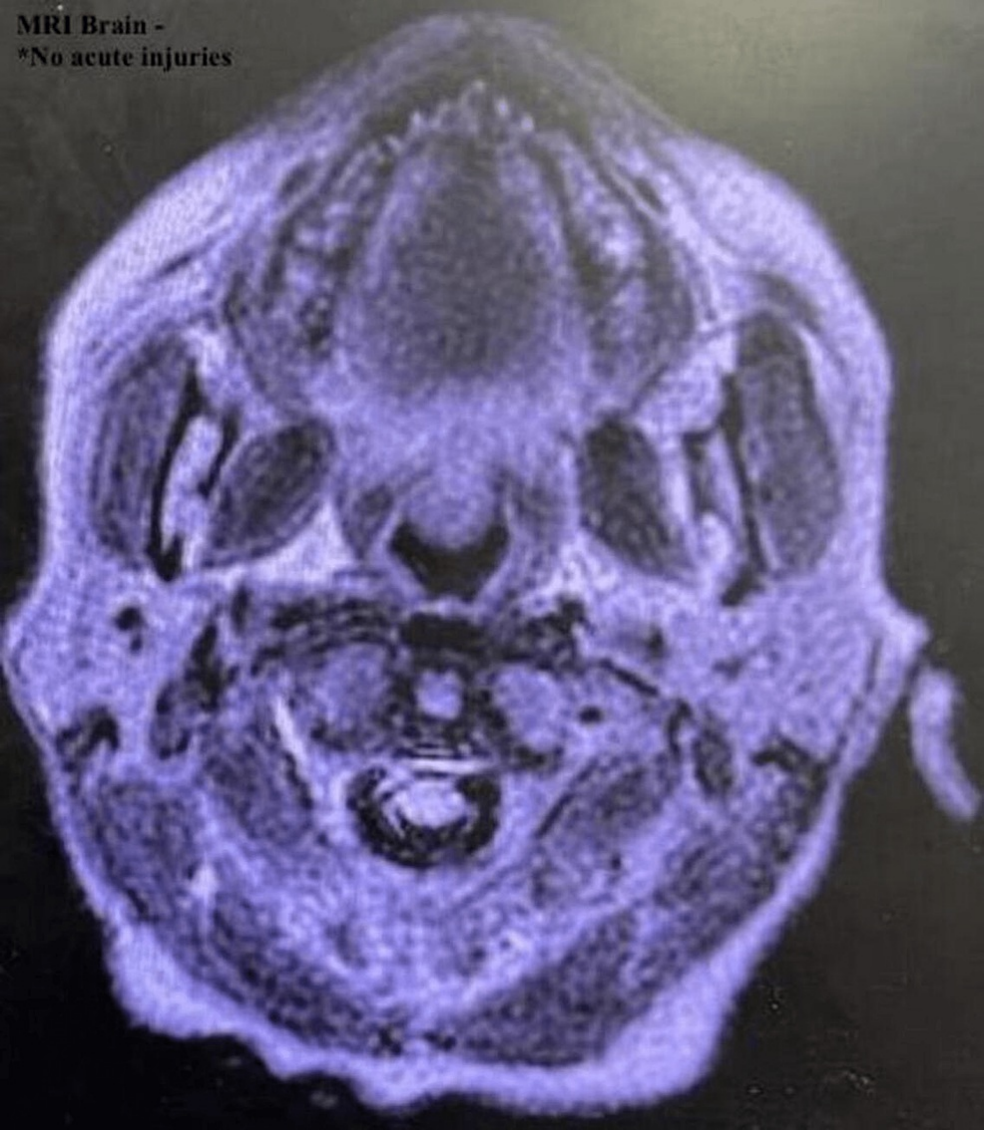
MRI of the brain MRI was indicated as a result of a fall during a syncopal episode. Exam date: 1/13/2023; 6:07 PM. Technique: MRI of the brain without intravenous contrast acquired at 1.5 tesla. MRI findings: No acute intracranial hemorrhage. No midline shift, herniation, or hydrocephalus. There were no abnormal foci on the susceptibility-weighted sequences. The major vascular flow voids were present. The mastoid air cells were clear. The osseous structures were unremarkable. The surrounding soft tissue structures were unremarkable.

The patient's inpatient care included consultations from both neurology and cardiology. The cause of the patient's fall and resultant head injury were determined to be caused by a combination of illicit drug use and a syncopal episode, as neurological causes were effectively ruled out. The patient was eventually discharged to a nursing facility. Recommendations were made to continue physical therapy outpatient, and a referral for substance abuse counseling to provide assistance with cessation was also made. 

## Discussion

As exemplified in this case, individuals who have continued to suffer from drug addictions that began during the height of the opioid epidemic are now members of the elderly adult population. Between the years 1992 and 2004, heroin was the injected drug most often reported at admission to drug treatment [8]. One of the consequences of heroin addiction is syncopal-induced falls. Falls are the number one cause of preventable injury in the elderly population. [1]. Common medical and non-medical risk factors for falls in the elderly typically include lower extremity weakness, vision problems, and home hazards. Medical professionals treating patients aged 65 and older are encouraged to perform annual evaluations inquiring whether or not their patients have had any falls or difficulty with walking or balance [9]. This line of questioning is used to determine if patients are at risk and also identify potentially modifiable risk factors for falls. Fall risk questionnaires that address substance abuse may be warranted so as to not exclude this growing subset in the elderly population. Research related to this population has shown that the number of older adults who will need treatment for a substance abuse problem will increase from approximately 1.7 million in the years 2000, and 2001 to approximately 4.4 million in 2020 [10]. Highlighting the various effects illicit drug use can have on the elderly population, which includes syncopal-related falls is essential to preventing the potential increase in the number of falls attributed to this cause.

Falls are the leading cause of injury-related morbidity and mortality among older adults in the United States. Although not all falls cause injury, many falls can cause bone fractures to the hands, wrist, or hip. Falls can also cause head injuries. Taking multiple medications to treat multiple medical conditions is called polypharmacy. Polypharmacy is also a risk factor for falls in the elderly. For example, elderly patients who are receiving treatment for atrial fibrillation, a common arrhythmia seen in elderly populations, may be taking blood thinners to decrease the risk of clot formation. Although taking blood thinners is a necessary treatment for their co-morbid conditions, blood thinners can have serious consequences including increased risk for bleeding after a fall. Falls can be prevented in many instances. Eliminating hazards in the home such as rugs or cords can reduce the risks of falls. Weight-based exercises have also been shown to decrease the risk of falls in the elderly. A potential addition to these common risk factors should be addressing substance abuse, specifically opioids like heroin, and how their use has been shown to lead to syncope which can increase the risk of a fall.

## Conclusions

The purpose of this case is to add awareness to the fact that the dynamics of the elderly population are changing, and the reasons attributed to falls may also be evolving. Falls are a well-known and well-studied cause of detrimental health outcomes in the elderly population. Many of the factors that contribute to falls have been addressed by preventative and educational awareness. On the other hand, limited research has been published regarding drug addiction as a risk factor for falls in the elderly population. In this case, heroin addiction specifically can lead to syncopal episodes and falls. As the elderly population, in general, continues to grow, and the populations of elderly individuals with substance abuse addictions also continue to grow, the likelihood of potential fall-related injuries may also be increasing. Addressing, correcting, or eliminating modifiable risk factors for falls in the elderly, can lead to a reduction of falls in this population. Professionals who are discussing ways to minimize modifiable fall risks in the elderly should also acknowledge how drug addiction can play a role in increasing falls as well. 
